# Development and validation of a latex-based lateral flow assay for the serological diagnosis of feline leishmaniosis caused by *Leishmania infantum*

**DOI:** 10.1186/s13071-026-07625-5

**Published:** 2026-08-12

**Authors:** Gabriel Puerta López, Sotiria Drakouli, Mireia Tarragó del Río, Irene Sánchez López, Diana Marteles-Aragüés, Sotirios Athanasiou, Sergio Villanueva-Saz

**Affiliations:** 1 Lateral Flow R&D Department, Uranovet S.L., Av. Santa Eulalia 2, 08520 Les Franqueses, Barcelona, Spain; 2 Lateral Flow Production Department, Uranovet S.L., Av. Santa Eulalia 2, 08520 Les Franqueses, Barcelona, Spain; 3 Animal Pathology Department, Veterinary Faculty and Instituto Agroalimentario de Aragón – IA2 (Universidad de Zaragoza-CITA), Miguel Servet 177, 50013 Saragossa, Spain; 4 Clinical Immunology Laboratory, Veterinary Faculty, University of Zaragoza, 50013 Saragossa, Spain

**Keywords:** Cat, ELISA, Lateral flow, *Leishmania infantum*, Rapid test

## Abstract

**Background:**

Feline leishmaniosis is a vector-borne disease caused by *Leishmania infantum* parasites and transmitted by infected phlebotomine sand flies. The infection is emerging and diagnostically challenging, partly because cats tend to exhibit lower and more variable antibody responses than dogs.

**Methods:**

In this study, a recombinant antigen-based diagnostic platform was developed and validated for the serological detection of *L. infantum* infection in cats, incorporating both enzyme-linked immunosorbent assay (ELISA) and lateral flow immunochromatographic assay (LFA) formats.

**Results:**

A total of 1118 feline serum samples were analyzed. Negative samples were confirmed by indirect immunofluorescence test and a recombinant antigen-based ELISA, while positive samples were defined using a dual-ELISA approach based on recombinant and crude antigens to establish a high-confidence reference set. The performance of a latex-based LFA was evaluated and compared with that of a conventional colloidal gold-based lateral flow assay. The latex-based LFA demonstrated high specificity (99.5%, 95% confidence interval [CI]: 98.9–99.8) and a markedly improved sensitivity (88.9%, 95% CI 78.4–95.4) compared with the colloidal gold-based assay (46.0%, 95% CI 33.4–58.9). Quantitative analysis of test line intensity revealed a mean 3.08-fold increase in signal with the latex-based system. Notably, a substantial proportion of positive samples missed by the colloidal gold assay were successfully identified by the latex-based platform.

**Conclusions:**

These findings indicate that the combination of recombinant antigen design with optimized latex-based detection significantly enhances the performance of lateral flow assays for feline leishmaniosis. This approach represents a promising strategy for the development of more sensitive and reliable point-of-care diagnostic tools in veterinary medicine.

**Graphical Abstract:**

**Figure:**
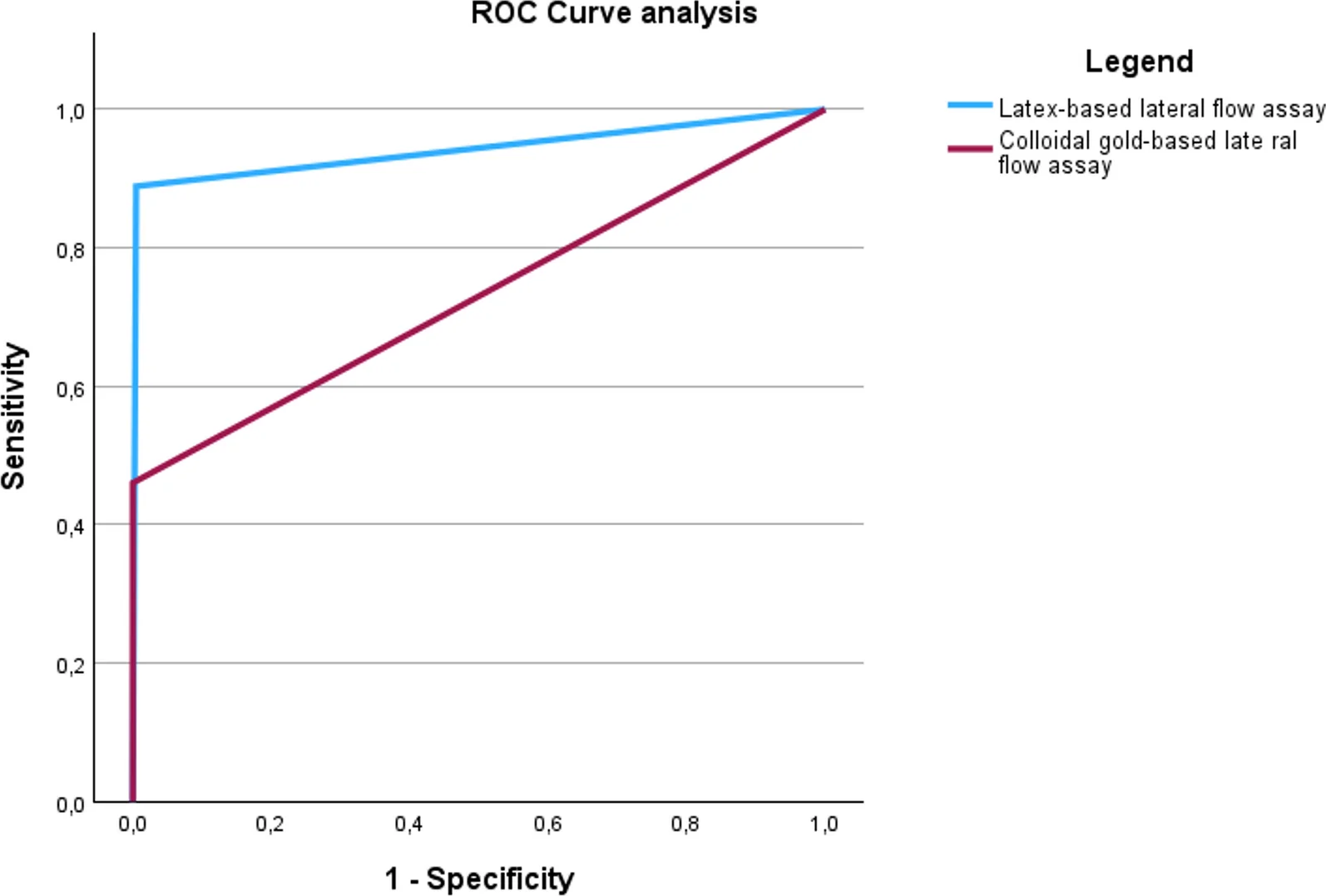

## Background

Leishmaniosis is a complex vector-borne disease caused by different species of the genus *Leishmania* that is endemic in 88 countries of southern Europe, Africa, Asia and South and Central America [1]. This group of zoonotic diseases affects humans as well as a broad spectrum of domestic [2–4] and wild vertebrates [5–7].

In the Mediterranean region of Europe, *Leishmania infantum* is the predominant etiological agent, and dogs have traditionally been regarded as the principal domestic reservoir for human infection [8]. However, cats are increasingly recognized as potential participants in the epidemiology of the disease in endemic areas, despite their apparently lower prevalence of infection compared with dogs [9, 10]. The epidemiological significance of cats remains incompletely defined. Current evidence does not support their role as primary reservoirs, yet their potential involvement as secondary hosts capable of contributing to transmission cycles cannot be excluded, particularly in environments with high vector density [3].

In dogs, the outcome of the parasite infection is strongly influenced by the host immune response, which may range from effective parasite control to progressive systemic disease [11]. Clinical expression varies widely, from subclinical infection to severe and potentially fatal leishmaniosis, and this heterogeneity has been linked mainly to differences in cell-mediated immunity, with host genetic background also contributing to susceptibility [12]. In general, resistance is associated with a parasite-specific T helper 1 (Th1) immune response characterized by activation of macrophages through cytokines such as Interferon-*γ* (IFN-γ), Interleukin-2, and tumor necrosis factor *α* [13–15], whereas disease progression is more often associated with a mixed Th1/Th2 immune response characterized by impaired or absent cell-mediated immunity, high parasite burden, and marked humoral responses with increased anti-*Leishmania* antibody production [16, 17]. In cats, the available evidence indicates that a comparable pattern of adaptive immunity also occurs following *L. infantum* exposure, with both humoral and cell-mediated responses being detectable in naturally exposed animals [18, 19] and the presence of circulating immune complexes associated with the disease [20]. However, feline responses appear to be generally less intense than those observed in dogs, and current data suggest that cats can mount a parasite-specific IFN-*γ* response, particularly in the absence of high antibody titers or detectable parasitemia.

Recent evidence indicates that cats develop an early Th1 immune response to *L. infantum*, which may contribute to more efficient control of infection and help explain the lower clinical severity observed relative to dogs [21]. Cats can harbor *L. infantum* and, under certain conditions, develop overt disease [20]; although infections are frequently subclinical and often associated with concurrent disorders or immunocompromised states [22, 23].

The clinical presentation of feline leishmaniosis (FeL) is variable and may be influenced by concurrent diseases, which can obscure the specific contribution of *L. infantum* to the observed abnormalities [24]. Skin and mucocutaneous lesions are the most commonly reported findings, followed by ocular manifestations such as conjunctivitis, blepharitis, keratoconjunctivitis, and uveitis. General signs including lymphadenomegaly, weight loss, anorexia, lethargy, fever, and splenomegaly may also occur. The main laboratory abnormalities described in affected cats are hyperglobulinemia, hypoalbuminemia, mild-to-moderate non-regenerative anemia, leukogram changes, thrombocytopenia, proteinuria, and renal azotemia [24, 25].

The diagnostic methods currently used to detect *Leishmania* infection in cats largely overlap with those employed in dogs [26]. Direct diagnostic approaches include polymerase chain reaction (PCR), cytology, histopathology, *Leishmania* spp.-specific immunohistochemistry, and parasite culture [20, 24]. Indirect diagnosis is primarily based on the serological detection of specific anti-*L. infantum* IgG antibodies using assays such as enzyme-linked immunosorbent assay (ELISA), immunofluorescence antibody test (IFAT), western blot (WB) [9], and direct agglutination test (DAT) [27]. Importantly, these serological methods must be specifically validated for feline use, as assays originally developed for dogs may perform suboptimally in cats. Such validation should include two main steps: first, the design, adaptation, and optimization of the assay for feline samples; and second, field validation in well-characterized cat populations [28]. Indeed, serological tests developed for dogs may yield negative results in truly seropositive cats, underscoring the need for feline-specific diagnostic validation. In dogs, the serological assays most commonly used in clinical practice to detect specific antibodies against *L. infantum* are IFAT, ELISA, and rapid immunochromatographic tests [29].

In areas where multiple *Leishmania* species coexist [30], serological diagnosis may be particularly challenging when conventional assays such as IFAT are employed, owing to the subjective interpretation of fluorescence and the potential for cross-reactivity within the genus. Moreover, because IFAT slides are generally based on whole promastigote forms as antigenic substrate, their specificity may be further limited. By contrast, recombinant proteins offer important diagnostic advantages. The use of species-specific recombinant antigens can improve specificity, reduce cross-reactivity, and enhance assay standardization, while lateral flow assays (LFA) also provide rapid and user-friendly testing. Together, these characteristics make recombinant antigen-based LFA and ELISA assays valuable tools for the diagnosis of leishmaniosis in complex epidemiological settings.

In recent years, recombinant antigens have emerged as a promising strategy to improve the accuracy of serological assays. Accordingly, the aim of the present study was to develop a novel recombinant antigen-based diagnostic platform, comprising ELISA and LFA formats, using half anti-feline IgG half-fragment conjugates to enhance the serological detection of *Leishmania* infection in cats.

## Methods

### Preparation of half-IgG fragments

Monoclonal mouse anti-feline IgG antibody (clone P9C2, IgG2b isotype) was produced in-house using standard hybridoma technology. The hybridoma was established on 16/08/2023 and selected based on its specific reactivity against feline IgG. Antibody specificity was verified through in-house functional testing within the lateral flow assay format. Controlled reduction was performed using tris(2-carboxyethyl)phosphine (TCEP) (Sigma-Aldrich, St. Louis, MO, USA) at a final concentration of 10 mM in phosphate-buffered saline (PBS) for 30 min under mild conditions. Following reduction, cysteine (Sigma-Aldrich, St. Louis, MO, USA) was added to quench the residual reducing agent and stabilize the generated half-IgG fragments. Purification was carried out by gel filtration chromatography using Sephadex G-25 columns (Cytiva, Uppsala, Sweden) previously equilibrated with 50 mM MES buffer (pH 6.0) (Sigma-Aldrich, St. Louis, MO, USA), in order to remove low-molecular-weight components and excess reagents. Purified fractions containing half-IgG fragments were collected and stored under appropriate conditions until further use. Purified fractions containing half-IgG fragments were collected based on chromatographic elution profiles and confirmed by SDS-PAGE analysis. Protein-containing fractions were further evaluated by UV absorbance at 280 nm, then pooled, aliquoted, and stored at − 20 °C, protected from repeated freeze–thaw cycles, until further use.

### Conjugation of half-IgG fragment to horseradish peroxidase

The purified half-IgG fragments were conjugated to horseradish peroxidase (HRP) (Thermo Fisher Scientific, Waltham, MA, USA) using a heterobifunctional maleimide–thiol crosslinking strategy. Briefly, HRP was activated with sulfo-SMCC (Thermo Fisher Scientific, Waltham, MA, USA), enabling covalent coupling to the thiol groups exposed on the reduced half-IgG fragments. The conjugation reaction was performed under controlled conditions to preserve both enzymatic activity and antibody functionality. Excess reagents and unreacted components were removed by gel filtration chromatography using PD-10 desalting columns (Cytiva, Uppsala, Sweden) based on size exclusion chromatography (SEC). This conjugation approach was based on established maleimide–thiol chemistries, as previously described for heterobifunctional crosslinking reactions [31]. Purified HRP-conjugated half-IgG fragments were collected and stored at − 20 °C in a 1:1 (v/v) solution of glycerol, until use in ELISA assays. The concentration of HRP-conjugated half-IgG fragments was estimated by absorbance at 280 nm following purification.

### Detection of L. infantum antibodies by a quantitative ELISA using a mixture of L. infantum recombinant proteins

Each plate was coated with 10 μg/mL of mouse anti-His-tag antibody in PBS (pH 7.4), and incubated overnight at 4 °C. Each plate was washed three times with 300 μL/well of PBS containing 0.05% Tween-20 (PBST), blocked with 300 μL/well of 1% bovine serum albumin (BSA) in PBS for 2 h at 37 °C, and then washed again with PBST. A 1:1 mixture of two custom-made *L. infantum* recombinant rK39 variants (Rekom Biotech, Granada, Spain), including the His-tag region, was used as the coating antigen. The variants differed in the extent of the K39 repeat region and represented the highest- and lowest-repeat variants. The antigen mixture was diluted in PBS to a final concentration of 0.25 μg/mL, added to the plates, and incubated overnight at 4 °C.

A 100-µl aliquot of feline serum, diluted 1:100 in PBST and 1% Casein (PBST-C), was added to each well. The plates were then incubated for 30 min at room temperature (RT), following which they were washed and 100 µL of half anti-feline IgG–HRP conjugate, diluted 1:10,000 in PBST-C was added. The plates were incubated for 10 min at room temperature (RT) and were washed again with PBST as described above. The substrate solution 3,3′,5,5′-tetramethylbenzidine (TMB) (Sigma-Aldrich, St. Louis, MO, USA) was added to each well and the reaction was allowed to develop for 10 min at RT in the dark. The reaction was stopped by adding 2.5 M sulfuric acid (H₂SO₄) (Sigma-Aldrich, St. Louis, MO, USA) to each well. Absorbance values were read at 450 nm in an automatic microELISA reader (Thermo Fisher Scientific Waltham, MA, USA). As a positive control (calibrator), each plate included serum from a cat from Spain diagnosed with FeL, confirmed by a positive *L. infantum* isolation in Novy–MacNeal–Nicolle (NNN) medium, and as a negative control, serum from a healthy, non-infected cat. An endpoint antibody titer was not determined in the calibrator serum. Instead, the serum was used as an internal normalization control and was arbitrarily assigned a value of 100 ELISA units. The calibrator serum was divided into single-use aliquots, stored frozen, and thawed only once, thereby avoiding repeated freeze–thaw cycles. Its stability during the study period was monitored indirectly through the optical density values obtained across independent assay plates. No formal long-term or accelerated stability study was performed. The same calibrator serum was used for all assays and plates, with a constant inter-assay variation of < 10%. The calibrator was used as an internal laboratory control and was not standardized against a certified external reference serum. Plates with an inter-assay variation of > 10% were discarded. All samples and controls were run in duplicate. The results were quantified as ELISA units (EU) compared to the positive control serum used as a calibrator and arbitrarily set at 100 EU. The cut-off was established at 10 EU (mean + 4 standard deviations [SD] of values from 50 indoor cats from northern Spain) and the results above this value were considered positive.

### Detection of L. infantum antibodies by an in-house single-dilution ELISA using crude antigen obtained from promastigote forms

The ELISA was performed on all sera as previously described [9], with some modifications. Briefly, each plate was coated with 20 µg/mL of crude antigen obtained from *L. infantum* promastigote forms (MHOM/MON-1/LEM 75) in 0.1 M carbonate/bicarbonate buffer (pH 9.6), and incubated overnight at 4 °C. A 100-µL aliquot of feline serum, diluted 1:100 in PBS containing 0.05% Tween 20 (PBST) and 1% dry skimmed milk (PBST-M), was added to each well. The plates were then incubated for 1 h at 37 °C in a moist chamber, after which they were washed and 100 µL of Protein A conjugated to horseradish peroxidase (Thermo Fisher Scientific Waltham, MA, USA) diluted 1:20,000 in PBST-M was added. The plates were incubated for 1 h at 37 °C in the moist chamber and then washed again with PBST and PBS as described above. The substrate solution (ortho-phenylenediamine) (Thermo Fisher Scientific Waltham, MA, USA) and stable peroxide substrate buffer (Thermo Fisher Scientific Waltham, MA, USA) were added to each well and the reaction was allowed to develop for 20 ± 5 min at RT in the dark. The reaction was stopped by adding 2.5 M H₂SO₄ (Sigma-Aldrich, St. Louis, MO, USA) to each well. Absorbance values were read at 492 nm in an automatic microELISA reader (Labsystems Multiskan, Midland, ON, Canada). As a positive control, each plate included serum from a cat from Spain diagnosed with FeL, confirmed by a positive *L. infantum* isolation using an NNN medium, and as a negative control, serum from a healthy, non-infected cat. The same calibrator serum was used for all assays and plates, with a constant inter-assay variation of <10%. Plates with an inter-assay variation of > 10% were discarded. All samples and controls were run in duplicate. Blank wells containing all assay reagents except feline serum were included on each plate. The mean blank OD was subtracted from the OD values of all samples and controls before calculation of ELISA units. Blank absorbance was consistently below 0.05. The results were quantified as ELISA units (EU) compared to the positive control serum used as a calibrator and arbitrarily set at 100 EU. The cut-off was established at 21 EU (mean + 4 standard deviations [SD] of values from 50 indoor cats from northern Spain) and the results above this value were considered to be positive.

### Detection of anti-Leishmania antibodies by an in-house two-fold serial dilution ELISA using crude antigen obtained from promastigote forms

All samples with an optical density (OD) equal or higher than 3 (signal saturation) were further analyzed using a two-fold serial dilution ELISA [20]. Serial dilutions started at 1:100 dilution and continued for 8 further dilutions in the same ELISA plate. To minimize inter-assay variability, all ELISA plates were performed on the same day. The results were quantified as EU related to a calibrator arbitrarily set at 100 EU with an OD of 1.00. The dilution at which the OD was closest to 1.00 was used for the calculation of the EU using the following formula: (sample OD/calibrator OD) × 100 × dilution factor.

### Latex beads’ conjugate preparation for LFA

Purified half anti-feline IgG fragments were conjugated to 300 nm carboxylated red latex beads (Estapor, 0.1% solid; Merck, Darmstadt, Germany) using a multi-step covalent coupling strategy. Beads were washed with 50 mM MES buffer (pH 6.0) and activated using 0.2 M EDC (Thermo Fisher Scientific, Waltham, MA, USA) and 0.2 M NHS (Thermo Fisher Scientific, Waltham, MA, USA) for 30 min at 37 °C, followed by centrifugation and washing. Hexamethylenediamine (Sigma-Aldrich, St. Louis, MO, USA) prepared in DMSO (Sigma-Aldrich, St. Louis, MO, USA) was introduced as a spacer molecule and incubated overnight at room temperature. After washing, the spacer was re-activated using EDC/NHS chemistry under the same conditions. Sulfo-SMCC (Thermo Fisher Scientific, Waltham, MA, USA) was then added and incubated for 60 min at 37 °C to enable thiol-reactive coupling. The conjugation protocol was optimized through systematic dose–response and time–response studies of the covalent coupling chemistry to identify conditions that provided the highest analytical signal while minimizing non-specific binding. The optimized formulation employed an antibody loading of 20 µg per mg of 300 nm carboxylated latex beads, followed by incubation overnight at RT. Unreacted sites were quenched using 5 mM cysteine (Sigma-Aldrich, St. Louis, MO, USA). The blocking strategy was also optimized by comparing several blocking agents, including BSA, casein, fish gelatin, ovalbumin, and a proprietary protein-based blocking formulation (Elipsis technology; Advanced Latex Conjugation Technology) based on a mixture of denatured protein fragments with defined molecular weight distribution. The selected blocking formulation provided the lowest non-specific binding while maintaining high analytical signal, consistent with efficient presentation of the immobilized antibodies. Detailed composition and fractionation parameters are proprietary and not disclosed.

The resulting conjugates were purified and formulated in a latex-stabilizing buffer containing 50 mM Tris, 0.5% casein, 1% Triton X-100 and 5% sucrose to prevent aggregation and maintain the particles’ stability. The direct physicochemical characterization of antibody orientation on the particle surface was not performed. However, the functional performance of the optimized conjugate, together with its stability and analytical performance, supports the suitability of the conjugation strategy for the intended diagnostic application.

### Membrane preparation

Nitrocellulose membranes Hi-Flow Plus HF090 (Merck KGaA, Darmstadt, Germany) were post-treated by immersion in a solution of 1 mM Tris (pH 8.2), 0.1% polyvinylpyrrolidone (PVP), and 0.1% Tween-20, followed by drying at 37 °C for 1 h. The effect of the treatment was evaluated by capillary flow analysis and image-based signal assessment. Capillary flow measurements showed a reduction in flow rate from 0.476 mm/s (untreated) to 0.206 mm/s (treated). ImageJ analysis indicated comparable test line intensities between untreated and treated membranes (104 vs. 103, respectively), while differences in background distribution resulted in improved signal-to-background separation under assay conditions. Test lines were applied by dispensing a mixture of three custom-made recombinant rK39 variants (Rekom Biotech, Granada, Spain) in a 2:1:1 ratio. The variants differed in the extent of the K39 repeat region of the *Leishmania* kinesin protein, with the 2:1:1 mixture comprising the highest-, intermediate-, and lowest-repeat variants, respectively. The relative proportion of each variant was determined empirically during assay optimization according to its individual immunoreactivity, while the inclusion of multiple variants was intended to broaden antigen representation in view of the reported sequence diversity of K39 repeat regions among *Leishmania* strains. Finally, control lines were applied using whole goat anti-mouse IgG (1 mg/mL). The test and control line were dispensed using a ratio of 1 uL/cm. Membranes were then dried at 37 °C for 1 h and stored in specific aluminum foils with desiccants and humidity indicators for keeping for keeping the humidity lower than 10%. Membrane treatment consisted of immersion in a solution of 1 mM Tris (pH 8.2), 0.1% PVP, and 0.1% Tween-20, followed by drying at 37 °C for 1 h. Membranes were then dried at 37 °C for 1 h and stored in sealed aluminum foil pouches with desiccants and humidity indicators, maintaining relative humidity below 10% until use.

### Conjugate pad preparation

Conjugate pads were fabricated from polyester fiber pads (Ahlstrom ReliaFlow^™^ 6614, Ahlstrom-Munksjö, Helsinki, Finland) with a basis weight of 120 g/m^2^ and a thickness of 0.38 mm. This material is a hydrophilic synthetic matrix designed for uniform fluid distribution and efficient release of dried conjugates in lateral flow assays. The conjugate formulation consisted of 50 mM Tris, 0.5% casein, 1% Triton X-100, and 5% sucrose, providing protein stabilization and surfactant-mediated reduction of non-specific interactions during drying and rehydration. Pads were loaded using a controlled dispensing system at a rate of 32 µL/cm. A custom 3D-printed platform, designed in-house and fabricated by additive manufacturing, was used to standardize conjugate pad preparation. The platform consists of a rigid grid-type support structure that ensures stable positioning of the conjugate pads during dispensing and drying, allowing controlled and reproducible processing conditions. After dispensing, pads were dried at 37 °C for 2 h under forced ventilation (100% airflow) and stored under desiccated conditions until assembly. Long-term stability of the prepared conjugate pads was evaluated by both accelerated and real-time studies. Accelerated testing at 45 °C for 7 days supported an extrapolated shelf life of 24 months. In addition, real-time stability testing demonstrated that conjugate pads stored at 2–30 °C for 24 months showed no significant reduction in test line intensity when evaluated with a common positive sample. Furthermore, negative samples remained negative throughout the study, with no increase in background signal or false-positive results.

### Characterization of the colloidal gold conjugate

Colloidal gold nanoparticles (40 nm, OD 1.0; BBI Solutions) were used for antibody conjugation. Conjugation was performed by passive adsorption. Briefly, the pH of the colloidal gold suspension was adjusted to 9.0 using K₂CO₃, and whole mouse anti-feline IgG was added at a final concentration of 20 µg/mL (gold solution at OD 1.0). The mixture was incubated for 1 h under gentle stirring at room temperature to allow antibody adsorption onto the particle surface.

Unbound antibody was removed by centrifugation at 8000 × g for 30 min. The pellet was resuspended in storage buffer consisting of 50 mM Tris, 0.5% casein, 1% Triton X-100, and 5% trehalose. The conjugate was subsequently blocked by incubation with casein at a final concentration of 0.5% for 30 min.

The conjugate was dispensed onto Ahlstrom-Munksjö conjugate pad material 6614 (WI, USA) at a controlled rate of 32 µL/cm and dried at 37 °C for 2 h with 100% ventilation prior to assembly into the lateral flow device.

### Lateral flow strip construction and testing

The final assembled lateral flow strip had a total length of 60 mm and consisted of a 20 mm sample pad, an 8 mm conjugate pad, a 25 mm nitrocellulose membrane, and a 20 mm absorbent pad. These components were overlapped on a plastic adhesive backing card. Overlap distances were defined as 6 mm (sample pad–conjugate pad), 2 mm (conjugate pad–nitrocellulose membrane), and 3 mm (nitrocellulose membrane–absorbent pad). Alignment was performed manually using a standardized assembly template to ensure consistent positioning of components across strips. Strips were cut to a width of 3.8 mm using a precision cutter (Kinematic Automation).

Assembled strips were stored individually in sealed aluminum foil pouches containing desiccant and humidity indicators, maintaining relative humidity below 10%. Maximum storage stability was evaluated up to 24 months at room temperature (20–25 °C).

Prior to testing, strips were equilibrated at room temperature (20–25 °C) for approximately 15–20 min after removal from storage to avoid moisture condensation associated with temperature differences between storage and ambient conditions. For testing, 10 µL of serum or whole blood was applied to the sample pad, followed by approximately 60 µL (2 drops) of sample diluent (PBS-T). After 10 min, results were interpreted visually. A positive result was defined as the appearance of a visible test line regardless of intensity. Weak or unclear bands were classified as indeterminate and retested when necessary.

Quantitative analysis of the lateral flow assay was performed using the ESEQuant LR3 lateral flow reader (Qiagen GmbH, Hilden, Germany). Test line intensity was automatically determined by instrument software and expressed as peak height values, which were used for statistical comparison between the latex bead- and colloidal gold-based assays. ImageJ software was used exclusively for the independent analysis of membrane background during the membrane treatment experiments and was not used for quantification of lateral flow assay signals.

The assembly process was optimized to maintain consistent flow rates, and ensure reproducible signal intensity as supported by the repeatability and reproducibility studies. The combination of hydrophilic membrane treatment, Ellipsis-conjugated latex beads and controlled conjugate pad deposition resulted in a high-performance platform suitable for the sensitive and reproducible detection of anti-*Leishmania* antibodies in feline serum. Following assembly, the strips were packaged individually in aluminum foil pouches containing a 1 g silica gel desiccant and stored at room temperature for up to 24 months.

### Robustness, repeatability, reproducibility and stability studies

The repeatability (intra-assay precision) of the LFA was evaluated by testing the same positive sample on eight independent lateral flow strips within a single analytical run. Reproducibility (inter-assay precision) was assessed by testing the same positive sample on eight separate days under identical experimental conditions. To assess assay performance near the limit of detection, a low-positive sample producing a signal at the threshold of visual interpretation was analyzed.

Stability studies conducted on the Latex *Leishmania* antibodies LFA demonstrated that the product maintained its performance characteristics under both accelerated aging and real-time storage conditions. Accelerated stability testing at 45 °C for 7 days supported an extrapolated shelf life of 24 months, while the ongoing real-time stability study confirmed that positive samples remained positive and negative samples remained negative after 24 months of storage at 2–30 °C.

### Sample collection

A total of approximately 1387 feline serum and blood samples were collected from November 2022 to April 2026 from different veterinary clinics across the Iberian Peninsula and the Canary Islands to investigate suspected cases of infection. Following quality control, 269 samples were excluded, including 210 due to insufficient sample volume for analysis and 59 due to severe hemolysis that could compromise the reliability of the serological assays. Consequently, 1118 samples were included in the final analysis.

A total of 71 samples were initially identified as positive using the recombinant antigen-based ELISA. These samples were subsequently analyzed using the crude antigen-based ELISA, which confirmed positivity in 63 samples. The remaining eight samples were positive only by the recombinant ELISA and negative by the crude antigen ELISA. Based on our predefined reference criterion, only samples that were positive by both ELISA methods were classified as true positives and included in the validation of the lateral flow assay, while the eight discordant samples were excluded from further analysis. Additionally, a panel of serum samples was included to evaluate potential cross-reactivity. This panel comprised samples from cats positive for *Dirofilaria immitis* antibodies, serum samples positive for *D. immitis* antigen, serum samples positive for feline leukemia virus (FeLV), and serum samples positive for feline immunodeficiency virus (FIV).

### LFA validation

Negative samples had previously undergone IFAT using a commercial kit (BioSystems S.A., Barcelona, Spain) as part of the routine diagnostic work-up before inclusion in this study. Because IFAT has lower analytical sensitivity than ELISA for the detection of anti-*Leishmania* antibodies in cats, all samples were subsequently evaluated using the recombinant antigen-based ELISA to minimize the risk of false-negative classification.

A total of 71 samples were initially reactive in the recombinant antigen-based ELISA. These samples were subsequently analyzed using the crude antigen-based ELISA, which confirmed positivity in 63 samples. The remaining eight samples were positive only in the recombinant antigen-based ELISA and negative in the crude antigen-based ELISA and were therefore excluded from the validation study. Only samples positive in both ELISA assays were classified as reference positives, whereas samples negative in the recombinant antigen-based ELISA were classified as reference negatives. Consequently, the final validation dataset comprised 63 positive and 1047 negative samples. This classification strategy was adopted to maximize the specificity of the reference-positive population while minimizing the possibility of false-negative classification associated with the lower analytical sensitivity of IFAT.

For lateral flow testing, 10 µL of serum or whole blood was applied to the sample pad, followed by approximately 60 µL (two drops) of PBS-T running buffer. Results were read after 10 min. A sample was considered positive when both the control line and a visible test line, irrespective of intensity, were present. Samples showing an absent control line were considered invalid and were retested.

### Statistical analysis

The composite reference classification was defined as follows: samples were classified as reference-positive when they yielded concordant positive results in both the recombinant antigen-based ELISA and the IFAT. Samples were classified as reference-negative when they yielded negative results in both IFAT and the recombinant antigen-based ELISA. The performance measures analyzed for each test were sensitivity, specificity, accuracy, area under the receiver operating characteristic curve (AUC-ROC), and positive and negative likelihood ratios were calculated using standard contingency tables. Agreement was assessed using Cohen’s coefficient (*κ*) by comparing the qualitative result of each LFA with the composite serological reference classification. These values were interpreted as follows: no agreement (*κ* < 0), slight agreement (0 < *κ* < 0.2), fair agreement (0.2 < *κ* < 0.4), moderate agreement (0.4 < *κ* < 0.6), substantial agreement (0.6 < *κ* < 0.8), and almost perfect agreement (*κ* > 0.8). AUC-ROC analyses were performed using the qualitative result of each LFA as a dichotomous test variable, coded as 0 for negative and 1 for positive. Because the LFA results were dichotomous, each AUC-ROC analysis was based on a single non-trivial operating point corresponding to the sensitivity and false-positive rate at the predefined qualitative decision threshold, together with the theoretical endpoints of the AUC-ROC space. Three categories were established to determine the accuracy of a diagnostic technique based on the AUC-ROC: high accuracy (0.9 < AUC-ROC ≤ 1), moderate accuracy (0.7 < AUC-ROC ≤ 0.9), and low accuracy (0.5 < AUC-ROC ≤ 0.7). Other parameters analyzed were positive predictive value (PPV) and negative predictive value (NPV). Both are measures of the effectiveness of a diagnostic test and depend on disease prevalence in the population. The seroprevalence in cats living in the Mediterranean basin is variable, depending on the region studied. In this study, PPV and NPV were calculated by taking into account the sensitivity and specificity obtained for each serological test evaluated, using reference seroprevalences reported for cats: areas with low-seroprevalence (8.9%) [27] and endemic areas with moderate to high-seroprevalence (32.1%) [32]. Finally, paired comparisons between the latex-based LFA and the colloidal gold-based LFA were performed using McNemar’s exact test, because both assays were evaluated on the same set of samples. Comparisons were performed separately for sensitivity, using only samples classified as positive by the composite serological reference standard, and for specificity, using only samples classified as negative. In addition, the overall proportion of correctly classified samples was compared between both assays. The 95% confidence intervals (CI) were calculated using the Wilson score method. A *p* value < 0.05 was considered significant. Statistical analyses were performed using IBM SPSS Statistics 30.0^®^ software (IBM, Armonk, NY, USA).

## Results

### Sample characterization

A total of 1118 feline serum samples passed quality control and were available for serological characterization. Of these, 1047 samples were negative in the recombinant antigen-based ELISA. A further 71 samples were initially reactive in the recombinant antigen-based ELISA and were subsequently analyzed using the crude antigen-based ELISA. Of these, 63 samples were also positive in the crude antigen-based ELISA, whereas 8 samples yielded discordant results and were excluded from the diagnostic validation.

Accordingly, the final validation dataset consisted of 1110 samples, including 1047 reference-negative samples and 63 reference-positive samples classified according to the composite reference standard based on the two ELISA assays.

**Fig. 1:**
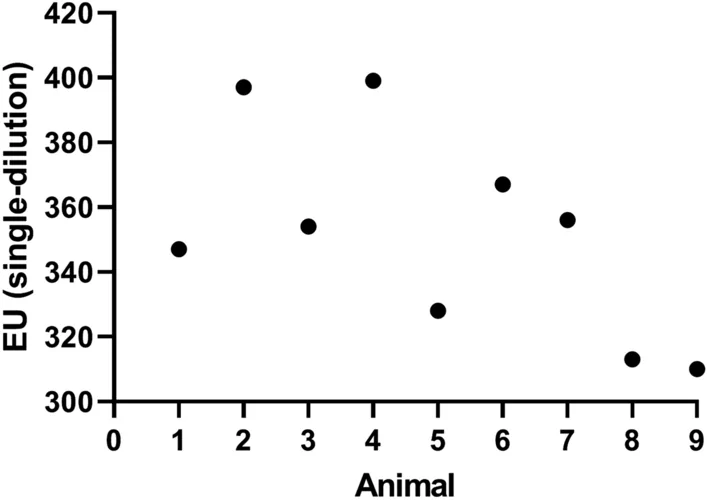
ELISA units (EU) obtained by the in-house single-dilution ELISA using crude *L. infantum* promastigote antigen in the nine seropositive cats with OD values greater than 3. Each dot represents one animal

**Fig. 2:**
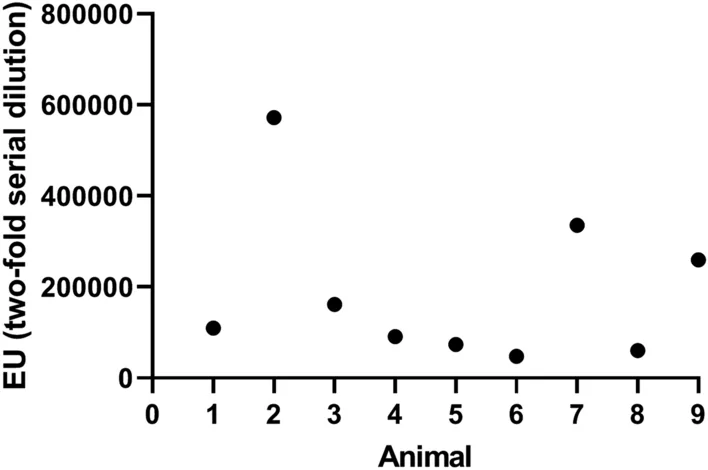
ELISA units (EU) obtained by the in-house two-fold serial dilution ELISA in the same nine seropositive cats with OD values greater than 3 in the single-dilution ELISA. The results show marked inter-individual variability and improved characterization of high-antibody samples compared with the single-dilution approach

Nine reference-positive samples exhibited optical density (OD) values greater than 3.0 in the single-dilution ELISA (Fig. 1). These samples were subsequently assessed by two-fold serial dilution, revealing greater variability in ELISA units (EU) (Fig. 2). This approach identified differences in estimated relative antibody reactivity that were not discernible under the saturated single-dilution conditions.

After the characterization by ELISA and LF, 95 positive serum samples for *D. immitis* antibodies, 10 positive serum samples for *D. immitis* antigen, 196 positive serum samples for FeLV and 271 positive serum samples for FIV were tested with the latex-based LFA. In all the cases there were negative results, showing that there was no cross-reactivity with the above pathogens.

### Performance of the latex-based LFA

Of the 1047 negative samples, the latex-based LFA correctly classified 1042 as negative, with 5 false-positive results, yielding a specificity of 99.5% (95% CI 98.9–99.8%). Of the 63 positive samples, the assay correctly identified 56, corresponding to a sensitivity of 88.9% (95% CI 78.4–95.4%) (Table 1). The accuracy was 98.8% (95% CI 98.1–99.4) and the agreement between the latex-based LFA and the composite serological reference classification was almost perfect (Cohen’s *κ* = 0.898). The latex bead-based assay yielded an LR+ of 186.2 and an LR− of 0.112, indicating that a positive result strongly increased the probability of infection, while a negative result substantially reduced it.

**Table 1:** Diagnostic performance of the latex-based LFA compared with IFAT and recombinant ELISA in feline serum samples

LFA test evaluated	IFAT and recombinant ELISA results		
	Positive	Negative	Total
Latex-based LFA			
Positive	56	5	61
Negative	7	1042	1049
Total	63	1047	1110
Colloidal gold-based LFA			
Positive	29	0	61
Negative	34	1047	1049
Total	63	1047	1110

The latex bead-based assay yielded an LR + of 186.2 and an LR − of 0.112, indicating that a positive result strongly increased the probability of infection, while a negative result substantially reduced it.

In a high-seroprevalence setting (32.1%), the PPV was 98.88% (95% CI 97.3–99.53%), whereas low-seroprevalence setting (8.9%), it was 94.8% (95% CI 88.3–97.8%. The NPV in a high-seroprevalence setting was 94.99% (95% CI 90.41–97.4%) and in low-seroprevalence setting was 98.9% (95% CI 97.9–99.5%). Finally, the accuracy in a high-seroprevalence setting was 96.1% (95% CI 94.8–97.2%), and in a low-seroprevalence setting, it was 98.6% (95% CI 97.7–99.2%).

### Performance of the colloidal gold-based LFA

Using the same panel of samples, the colloidal gold-based LFA correctly classified all 1047 negative samples as negative, resulting in a specificity of 100% (95% CI 98.9–99.8%). However, only 29 of the 63 positive samples were correctly identified, corresponding to a sensitivity of 46.0% (95% CI 33.4–58.9%) (Table 2). Accuracy was 96.9% (95% CI 95.8–97.9%), and the agreement between the colloidal gold-based LFA and the composite serological reference classification was substantial (Cohen’s *κ* = 0.617). The colloidal gold-based lateral flow assay showed a positive likelihood ratio approaching infinity due to the absence of false-positive results, whereas the negative likelihood ratio was 0.54.

**Table 2:** Quantitative comparison of test line signal intensity between latex-based and colloidal gold-based assays

Sample ID	Pick height		Ratio LB/CG
	Colloidal gold-based LFA	Latex-based LFA	
189	43.65	57.68	1.32
288	156.03	552.85	3.54
579	227.94	303.56	1.33
52	95.52	415.39	4.35
183	83.46	317.44	3.80
79	51.14	126.72	2.48
239	241.58	463.34	1.92
244	162.75	397.71	2.44
249	191.91	628.29	3.27
251	139.60	376.65	2.70
586	35.28	333.62	9.46
34	385.11	762.96	1.98
247	343.53	651.12	1.90
584	314.48	819.78	2.61
			3.08
			Ratio mean

Quantitative analysis of test line signal intensity obtained from latex-based (LB) and colloidal gold-based (CG) lateral flow assays (LFA) using a Qiagen ESEQuant LR3 reader. Data are shown for a subset of 14 representative positive samples. Peak height values for each system and the corresponding LB/CG signal ratio are presented. The mean signal ratio (3.08) reflects the enhanced signal generation capacity of the latex-based assay

Analysis of the 34 false-negative samples obtained with the colloidal gold-based lateral flow assay showed that the majority (approximately 90%) had low ELISA reactivity to recombinant antigens, with signal ratios ranging from 0.190 to 0.902. Only a small number of false-negative samples showed intermediate reactivity, with a maximum ratio of 1.318. In contrast, samples correctly identified as positive by the assay exhibited substantially higher ELISA ratios, ranging from 1.718 to 4.650. These results indicate that false-negative results were mainly associated with low antibody levels near the assay detection limit.

In a high-seroprevalence setting (32.1%), the PPV was 100% (95% CI 88.1–100.0%), whereas in a low-seroprevalence setting (8.9%), it was 100% (95% CI 88.1–100%). The NPV in a high-seroprevalence setting was 79.7% (95% CI 75.7–83.1%) and in low-seroprevalence setting was 95.0% (95% CI 93.8–96.0%). The colloidal gold-based lateral flow assay showed a positive likelihood ratio approaching infinity due to the absence of false-positive results, whereas the negative likelihood ratio was 0.54.

Finally, the accuracy in a high-seroprevalence setting was 82.7% (95% CI 80.3–84.9%), and in a low-seroprevalence setting, this diagnostic measure was 95.2% (95% CI 93.8–96.4%).

AUC-ROC analysis based on the dichotomous qualitative outcomes of the two LFAs yielded an AUC-ROC of 0.942 for the latex-based assay and 0.730 for the colloidal gold-based assay. These values corresponded to the operating point defined by the predefined positive/negative interpretation of each test. Overall, the latex-based assay demonstrated a 1.93-fold increase in sensitivity compared with the colloidal gold-based system (88.9% vs. 46.0%). Notably, a substantial number of positive samples that were not detected by the colloidal gold-based assay were successfully identified using the latex-based system.

### Comparison of diagnostic performance between latex-based and colloidal gold-based LFAs

Paired comparison of the two LFAs was performed using McNemar’s exact test. Among the 63 positive samples, 29 were positive by both assays, 27 were positive only by the latex-based LFA, none was positive only by the colloidal gold-based LFA, and seven were negative by both assays. McNemar’s exact test demonstrated that the latex-based LFA had a significantly higher sensitivity than the colloidal gold-based assay (*p* < 0.001).

Among the 1047 negative samples, five were positive only by the latex-based LFA and 1042 were negative by both assays, whereas no sample was positive only by the colloidal gold-based LFA. The difference in specificity between the two assays did not reach statistical significance by McNemar’s exact test (*p* = 0.063).

When the overall classification performance was compared, the latex-based LFA correctly classified 1098 of 1110 samples, whereas the colloidal gold-based LFA correctly classified 1076 of 1110 samples. McNemar’s exact test showed that the overall proportion of correctly classified samples was significantly higher for the latex-based LFA than for the colloidal gold-based assay (*p* < 0.001).

### Quantitative analysis of signal intensity

**Fig. 3:**
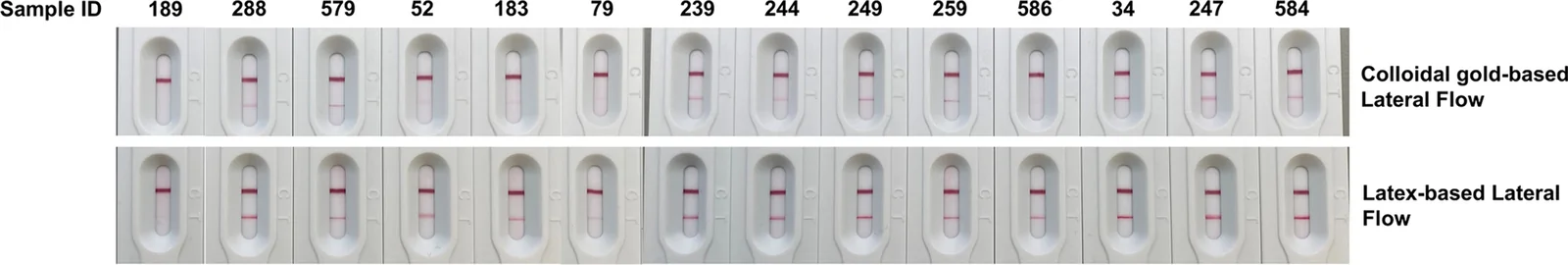
Quantitative comparison of test line signal intensity between latex-based and colloidal gold-based LFAs. Test line signal intensity was measured for 14 representative positive samples using a Qiagen ESEQuant LR3 reader. The latex-based assay (LB) consistently produced higher signal intensities compared to the colloidal gold-based assay (CG). Individual values are shown for each sample, together with the calculated LB/CG signal ratios. The mean signal ratio (3.08) highlights the enhanced signal generation capacity of the latex-based system

Quantitative analysis of test-line signal intensity was performed on a subset of 14 reference-positive samples selected to represent a broad range of ELISA optical density (OD) values, including samples close to the diagnostic cut-off and samples with high antibody levels (OD > 3.0). Test-line intensities were measured using the Qiagen ESEQuant LR3 lateral flow reader (Qiagen GmbH, Hilden, Germany) (Fig. 3). This subset was selected to evaluate signal generation across different levels of serological reactivity and to investigate whether differences in signal intensity could help explain the improved diagnostic sensitivity observed for the latex bead-based assay.

Quantitative signal intensities obtained from the 14 paired samples were significantly higher for the latex bead-based assay than for the colloidal gold-based assay (paired *t*-test: *t* = 7.03, df = 13, *P* < 0.0001). Across all paired samples, the latex bead conjugate produced mean test-line signal that was 3.08-fold higher than the colloidal gold conjugate.

Correlation analysis between ELISA optical density (OD) values and LFA signal intensities showed a weak and non-significant association for both detection systems. For the latex bead-based assay, Pearson’s correlation coefficient was *r* = 0.306 (*p* = 0.287) and Spearman’s *ρ* = 0.108 (*p* = 0.713), while the colloidal gold assay showed *r* = 0.101 (*p* = 0.732) and *ρ* = − 0.064 (*p* = 0.828). These results indicate that LFA signal intensity is not strictly proportional to ELISA OD values across the full range of antibody reactivity. False-negative results were mainly observed in samples with ELISA OD values close to the diagnostic cut-off, whereas samples with higher OD values were more consistently detected, particularly in the latex bead-based assay. The LFA signal reflects antigen–antibody interaction under flow conditions rather than direct proportionality to ELISA OD, consistent with the heterogeneous nature of antibody binding in field samples.

### Limited analytical validation

The repeatability (intra-assay precision) of the LFA was evaluated by testing the same positive sample on eight independent lateral flow strips within a single analytical run. The intensity of the test line was quantified, yielding a coefficient of variation (CV) of 8.39%, indicating good repeatability of the assay. Reproducibility (inter-assay precision) was assessed by testing the same positive sample on eight different days under the same experimental conditions. Quantification of the test line resulted in a CV of 9.61%, demonstrating good reproducibility between assay runs.

To assess the assay performance near the limit of detection, a low-positive sample producing a signal at the threshold of visual interpretation was analyzed. Quantification of the test line yielded a CV of 12.31%, indicating acceptable precision at the lowest detectable signal level.

### Membrane modification process

**Fig. 4:**
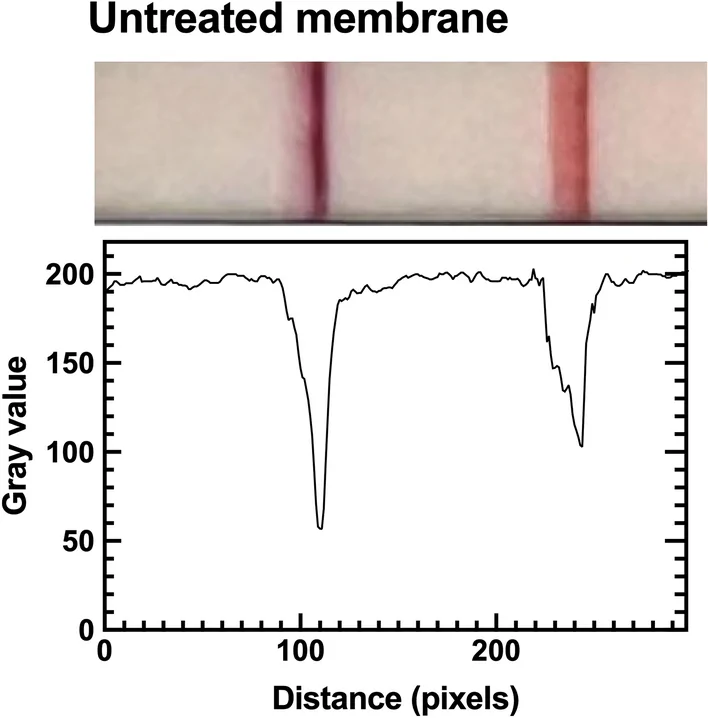
Representative image of a lateral flow strip prepared using an untreated nitrocellulose membrane. The intensity profile was obtained by grayscale image analysis using ImageJ. A line scan was performed across the membrane, and gray values were plotted as a function of distance (pixels). The first peak corresponds to the test line, and the second peak corresponds to the control line. Signal intensity was quantified from the minimum gray value at each line, while the background was determined from adjacent membrane regions

**Fig. 5:**
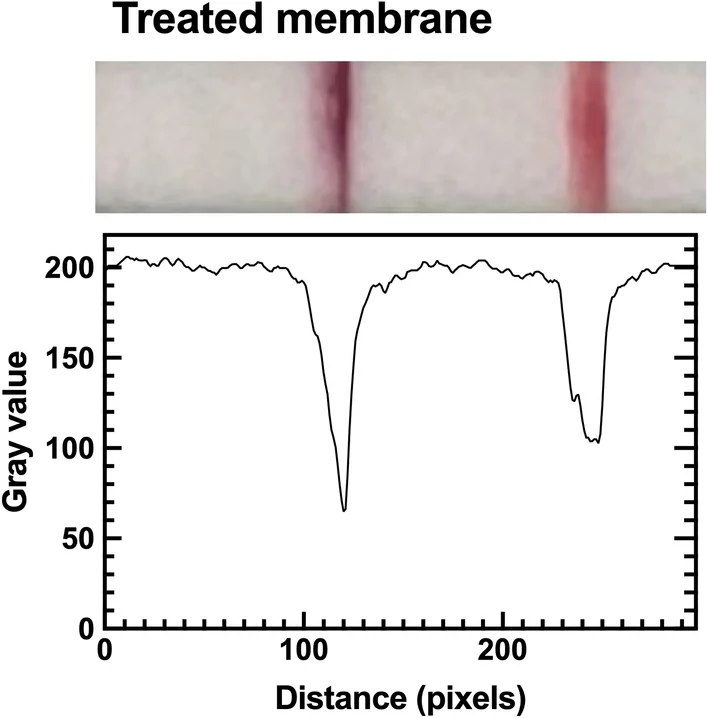
Representative image of a lateral flow strip prepared using the treated nitrocellulose membrane. The intensity profile was generated by grayscale image analysis using ImageJ as described for Fig. 4. The first peak corresponds to the test line, and the second peak corresponds to the control line. Comparison with the untreated membrane illustrates the effect of membrane treatment on background intensity while maintaining comparable test-line signal

Capillary flow measurements demonstrated a reduction in flow rate from 0.476 mm/s in untreated membranes to 0.206 mm/s in treated membranes, indicating a moderated flow profile. ImageJ-based analysis showed that test line intensities remained comparable between treated and untreated membranes (104 vs. 103, respectively), while background signal and contrast distribution were modified, resulting in improved signal-to-background separation under assay conditions (Figs. 4 and 5).

### Robustness, repeatability, reproducibility and stability studies

The control line intensity was quantified, yielding a coefficient of variation (CV) of 8.39%, which indicated good assay repeatability. For reproducibility, quantification of the control line yielded a CV of 9.61%, demonstrating good inter-assay precision. At the lowest detectable signal level, the control line intensity showed a CV of 12.31%, indicating acceptable precision near the assay’s limit of detection. Finally, no significant reduction in test line intensity was observed compared with the initial evaluation, indicating that the kit remained stable throughout its intended shelf life.

## Discussion

FeL remains an under-recognized and diagnostically challenging condition, largely due to the lower and more variable humoral response in cats than in dogs [25]. This has direct consequences for serological testing, since assays optimized for canine samples may show reduced sensitivity when applied to cats. In this context, the development of diagnostic tools specifically adapted to feline immunobiology is particularly relevant.

In the present study, we developed and validated a recombinant antigen-based serological platform for the detection of anti-*Leishmania infantum* antibodies in cats, including both ELISA and lateral flow formats. One of the strengths of the study was the use of a composite reference approach to define the validation panel, whereby positive samples were required to react in both recombinant and crude ELISAs, while negative samples were supported by IFAT and recombinant ELISA results.

Given the absence of a definitive gold standard for FeL, a composite serological reference classification was adopted to define the sample panel used for LFA evaluation. Samples classified as reference-positive were required to show concordant reactivity in both the recombinant antigen-based ELISA and the crude promastigote antigen-based ELISA, whereas reference-negative samples were negative by IFAT and the recombinant antigen-based ELISA. This pragmatic approach allowed consistent classification of the available serum samples, but it does not provide independent confirmation of infection and may be affected by incorporation bias and imperfect serological sensitivity. This strategy preferentially selected samples with clearly concordant positive or negative serological profiles. Therefore, the resulting panel may not fully represent the spectrum of antibody responses encountered in routine clinical populations, particularly low-positive, borderline, or discordant samples.

This approach is consistent with those used in epidemiological studies of FeL, which typically combine different confirmatory techniques to identify *L. infantum* infection [9]. In our study, the reference methods included the most commonly used serological techniques in dogs [29] and cats [33, 34], such as IFAT and ELISA, as these are the only confirmatory methods capable of detecting the humoral immune response through the production of anti-*Leishmania* antibodies, unlike other techniques such as PCR or parasitological methods or PCR, which detect the parasite or its genetic material [35].

Specifically, the latex format reached a sensitivity of 88.9%, compared with 46.0% for the colloidal gold system, while maintaining very high specificity. This difference is especially important (88.9% for latex format versus 46.0% for colloidal gold system; *p* < 0.001) in the detection of anti-*Leishmania* antibodies in cats, where low antibody titers may produce weak or undetectable signals in conventional rapid tests [33]. The improved analytical performance of the latex-based system was consistent with the reader-based assessment of signal intensity, which showed a mean 3.08-fold increase in the test-line signal relative to that of the colloidal gold assay.

Several factors may have contributed to the improved performance of the latex-based platform. The higher signal intensity observed for the latex bead-based assay likely contributed to its improved detectability and overall sensitivity compared with the colloidal gold system. The use of half anti-feline IgG fragments may have reduced steric hindrance and facilitated antigen–antibody interaction. However, no direct experimental evidence was obtained to confirm antibody orientation, and the contribution of individual system components cannot be distinguished in this study.

An important practical finding was that 34 positive samples undetected by the colloidal gold-based assay were successfully identified by the latex-based test. This observation has potential clinical and epidemiological relevance. In endemic settings, underdetection of cats with low-level antibody responses may lead to underestimation of exposure or infection rates. A more sensitive point-of-care assay could therefore improve screening strategies, support case recognition in animals with compatible clinical signs, and strengthen seroepidemiological studies. At the same time, serological results should still be interpreted alongside clinical findings and, when appropriate, complementary tests, since antibody detection does not by itself establish disease causality.

The use of recombinant antigens is another relevant strength of the platform. Compared with crude antigen preparations or whole-parasite substrates, recombinant proteins offer improved standardization and may reduce non-specific reactivity. This is particularly valuable in areas where different *Leishmania* species may coexist or where broad serological cross-reactivity can complicate interpretation [36]. The combination of recombinant antigen design with an optimized latex-based readout therefore represents a rational approach to enhancing diagnostic performance.

Our study highlights marked inter-individual variability in antibody levels among cats at the time of diagnosis, consistent with previous findings in dogs [37] and in a cat with leishmaniosis during follow-up [20]. This variability was evident even among serum samples with very high antibody levels, highlighting the analytical value of two-fold serial dilution. Within the limited subset of nine highly reactive samples, serial dilution revealed differences in relative antibody reactivity despite similarly high OD values at the initial 1:100 dilution. This exploratory finding illustrates the limited ability of the single-dilution assay to discriminate among samples once the signal approaches saturation and enabled further analytical characterization of antibody reactivity under these conditions. However, it should not be interpreted as evidence of the overall magnitude of inter-individual variability in the wider feline population. Moreover, the present study did not assess whether serial dilution improved diagnostic classification, sensitivity, or specificity.

Ideally, validation of a diagnostic assay for FeLs should include cats characterized by independent molecular or parasitological methods, including PCR, cytology, culture, histopathology, or immunohistochemistry. However, obtaining large panels of fully characterized clinical cases is particularly difficult in cats, because naturally occurring clinical FeL is less frequently documented than canine leishmaniosis and complete clinical, molecular, and parasitological characterization is not always available. Moreover, epidemiological studies in cats commonly rely on combinations of serological and molecular techniques, or on serology alone, depending on the study design and sample availability. Importantly, PCR and serology do not always identify the same infected animals. Previous studies have reported only fair agreement between blood qPCR and IFAT or ELISA, and slight agreement between blood qPCR and WB, suggesting that molecular and serological methods may reflect different biological aspects of infection [9]. The absence of an independent gold-standard reference method for the diagnosis of canine leishmaniosis is a recognized limitation in diagnostic accuracy studies [38]. Therefore, although the lack of an independent molecular or parasitological reference standard remains a limitation of the present study in FeL, the use of a stringent dual-ELISA serological definition was considered appropriate for evaluating an antibody-detection assay.

Some limitations should nevertheless be acknowledged. First, the reference classification was composite and serology-based rather than derived from a single independent gold-standard technique. Because recombinant antigen-based serology formed part of the validation strategy, some degree of incorporation bias cannot be excluded. Moreover, the reference classification procedure was not applied symmetrically to all samples. Samples positive by the recombinant antigen-based ELISA were subsequently evaluated using the crude promastigote antigen-based ELISA, whereas samples classified as reference-negative on the basis of negative IFAT and recombinant ELISA results were not systematically tested with the crude ELISA. This differential verification procedure may have introduced classification bias, as some samples assigned to the reference-negative group could potentially have exhibited positive or borderline reactivity in the crude antigen-based assay. Second, the number of reference-positive samples was modest, which may have limited the precision of the sensitivity estimate. In addition, the study lacked an independent external validation cohort. Although the assay was evaluated using a large and geographically diverse sample set collected from multiple veterinary clinics across the Iberian Peninsula and the Canary Islands, all samples were processed within the same study framework. Validation in independent sample populations will therefore be necessary to further confirm the diagnostic performance and generalizability of the assay. Third, quantitative signal analysis was restricted to a subset of samples. Specifically, the quantitative comparison between latex beads and colloidal gold was based on only 14 paired samples because the limited sensitivity and dynamic range of the image quantification system did not allow reliable measurement of the weaker signals generated by colloidal gold in all samples. To ensure a direct and methodologically consistent comparison between the two labeling systems, only samples yielding measurable signals with both assays were included in the quantitative analysis. Although this reduced the sample size, the resulting paired dataset enabled a consistent comparison of signal intensities between the two detection systems. Despite these limitations, the latex-based assay produced only a small number of false-positive results, while maintaining high sensitivity. This performance profile may be particularly advantageous in screening settings, where minimizing false-negative results is a primary objective.

Overall, the present findings indicate that integrating recombinant antigens with an optimized latex-based lateral flow system may improve the serological detection of *L. infantum* infection in cats. The marked increase in sensitivity, together with the maintenance of high specificity, suggests that this approach may overcome some of the main limitations of conventional colloidal gold rapid tests in feline samples. These results support the potential value of this platform as a more reliable point-of-care tool for the diagnosis and surveillance of FeL.

## Conclusions

The present study describes the development and validation of a recombinant antigen-based diagnostic platform for the serological detection of *L. infantum* infection in cats, incorporating both ELISA and LFA formats. The latex-based lateral flow assay demonstrated high specificity and a markedly improved sensitivity compared with a conventional colloidal gold-based system, along with significantly enhanced signal intensity. These findings underscore the importance of optimizing both antigen design and detection chemistry in the development of diagnostic tools for feline samples, in which antibody responses are often lower and more variable. Overall, the results indicate that the proposed approach can improve the detection of anti-*Leishmania* antibodies, including in samples that may be missed by conventional assays, with potential relevance for both clinical diagnosis and epidemiological surveillance of FeL. In addition, the integration of recombinant antigens with advanced latex-based conjugation strategies represents a promising avenue for the development of more sensitive and reliable point-of-care diagnostic tests in veterinary medicine.

## Data Availability

Data supporting the main conclusions of this study are included in the manuscript.

## Abbreviations

AUC-ROC: Area under the receiver operating characteristic curve
CI: Confidence interval
DAT: Direct agglutination test
ELISA: Enzyme-linked immunosorbent assay
EU: ELISA units
IgG: Immunoglobulin G
IFAT: Immunofluorescence antibody test
*κ*: Cohen’s *κ* coefficient
LFA: Lateral flow assay
NPV: Negative predictive value
OD: Optical density
PCR: Polymerase chain reaction
PPV: Positive predictive value
Th: T helper cells

## References

[1] Alvar J, Yactayo S, Bern C. Leishmaniasis and poverty. Trends Parasitol. 2006;22:552–7. 10.1016/j.pt.2006.09.004. 17023215

[2] Schäfer I, Schmidt A, Gräßer F, Schieszler A, Aupperle-Lellbach H, Loesenbeck G, et al. Feline leishmaniosis with focus on ocular manifestation: a case report. Parasit Vectors. 2023;16:161. 10.1186/s13071-023-05741-0. PMC10176290 37173777

[3] Marteles D, Martínez MV, Fernández A, Riera C, Fisa R, Roca-Geronès X, et al. Assessment of the exposure to *Phlebotomus perniciosus* and the presence of anti-*Leishmania infantum* antibodies in stray cats in an endemic region of Spain, and their potential correlation with environmental factors. Vet Q. 2024;44:1–8. 10.1080/01652176.2024.2421308. PMC11544736 39508077

[4] Murillo A, Lebrero ME, Valdés M, Ramos J, Villanueva-Saz S, Davies JE, et al. Post-surgical seroreversion in a case of equine cutaneous leishmaniosis by *Leishmania infantum*. Vet Res Commun. 2026;50:91. 10.1007/s11259-025-11031-0. PMC12783306 41504811

[5] Del Carmen Aranda M, Villora J, Giner J, Fernández A, Quilez P, Davies JE, et al. A longitudinal study on the detection of anti-*Leishmania* antibodies in a captive European mink (*Mustela lutreola*) population and their correlation with serum protein electrophoresis. Res Vet Sci. 2025;185:105541. 10.1016/j.rvsc.2025.105541. 39837108

[6] Villanueva-Saz S, Lebrero ME, Giner J, Lillo SM, Guerra R, Roca-Geronès X, et al. Naturally acquired visceral leishmaniosis in a captive white-naped mangabey (*Cercocebus lunulatus*) in Spain. Vet Res Commun. 2025;49:318. 10.1007/s11259-025-10894-7. PMC12449416 40971031

[7] Barbero-Moyano J, Marteles D, Cano-Terriza D, Villanueva-Saz S, Gonzálvez M, Quilez P, et al. Serosurveillance of *Leishmania infantum* in zoo-kept animals in Spain. Zoonoses Public Health. 2026;73:65–73. 10.1111/zph.70024. PMC12765971 41243428

[8] Baneth G, Thamsborg SM, Otranto D, Guillot J, Blaga R, Deplazes P, et al. Major parasitic zoonoses associated with dogs and cats in Europe. J Comp Pathol. 2016;155:S54–74. 10.1016/j.jcpa.2015.10.179. 26687277

[9] Alcover MM, Basurco A, Fernandez A, Riera C, Fisa R, Gonzalez A, et al. A cross-sectional study of *Leishmania infantum* infection in stray cats in the city of Zaragoza (Spain) using serology and PCR. Parasit Vectors. 2021;14:178. 10.1186/s13071-021-04682-w. PMC7992781 33766113

[10] Villanueva-Saz S, Martínez M, Nijhof AM, Gerst B, Gentil M, Müller E, et al. Molecular survey on vector-borne pathogens in clinically healthy stray cats in Zaragoza (Spain). Parasit Vectors. 2023;16:428. 10.1186/s13071-023-06046-y. PMC10662132 37986028

[11] Hosein S, Blake DP, Solano-Gallego L. Insights on adaptive and innate immunity in canine leishmaniosis. Parasitology. 2017;144:95–115. 10.1017/S003118201600055X. PMC5264656 27094260

[12] Pinelli E, Killick-Kendrick R, Wagenaar J, Bernadina W, del Real G, Ruitenberg J. Cellular and humoral immune responses in dogs experimentally and naturally infected with *Leishmania infantum*. Infect Immun. 1994;62:229–35. 10.1128/iai.62.1.229-235. PMC186091 8262632

[13] Barbiéri CL. Immunology of canine leishmaniasis. Parasite Immunol. 2006;28:329–37. 10.1111/j.1365-3024.2006.00840.x. 16842269

[14] Maia C, Campino L. Cytokine and phenotypic cell profiles of *Leishmania infantum* infection in the dog. J Trop Med. 2012. 10.1155/2012/541571. PMC3154519 21845197

[15] Martínez-Orellana P, Marí-Martorell D, Montserrat-Sangrà S, Ordeix L, Baneth G, Solano-Gallego L. *Leishmania infantum*-specific IFN-γ production in stimulated blood from dogs with clinical leishmaniosis at diagnosis and during treatment. Vet Parasitol. 2017;248:39–47. 10.1016/j.vetpar.2017.10.018. 29173539

[16] Chamizo C, Moreno J, Alvar J. Semi-quantitative analysis of cytokine expression in asymptomatic canine leishmaniasis. Vet Immunol Immunopathol. 2005;103:67–75. 10.1016/j.vetimm.2004.08.010. 15626462

[17] Carrillo E, Moreno J. Cytokine profiles in canine visceral leishmaniasis. Vet Immunol Immunopathol. 2009;128:67–70. 10.1016/j.vetimm.2008.10.310. 19054573

[18] Priolo V, Martínez-Orellana P, Pennisi MG, Masucci M, Prandi D, Ippolito D, et al. *Leishmania infantum*-specific IFN-γ production in stimulated blood from cats living in areas where canine leishmaniosis is endemic. Parasit Vectors. 2019;12:133. 10.1186/s13071-019-3386-y. PMC6434818 30909952

[19] Lode JVL, Ito LTS, Umino GM, de Lima VMF. IL-12 and IFN-γ in response to *Leishmania infantum* antigens in felines from an endemic area for visceral leishmaniasis. Parasite Immunol. 2025;47:e70021. 10.1111/pim.70021. PMC12344394 40799008

[20] González A, Parody N, Renshaw A, Navarro G, Montañés L, Lebrero ME, et al. Detection of circulating immune complexes in feline leishmaniosis: first evidence and diagnostic implications. Vet Res Commun. 2026;50:185. 10.1007/s11259-026-11120-8. PMC12953345 41770288

[21] Louzada-Flores VN, Palazzo N, Gernone F, Uva A, Barrs VR, Mendoza-Roldan JA, et al. Cytokine gene expression in feline leishmaniasis: why cats might be less clinically affected than dogs. Parasit Vectors. 2026;19:127. 10.1186/s13071-026-07285-5. PMC13011642 41691321

[22] Brianti E, Celi N, Napoli E, Abbate JM, Arfuso F, Gaglio G, et al. Treatment and long-term follow-up of a cat with leishmaniosis. Parasit Vectors. 2019;12:121. 10.1186/s13071-019-3388-9. PMC6434852 30909954

[23] Garcia-Torres M, López MC, Tasker S, Lappin MR, Blasi-Brugué C, Roura X. Review and statistical analysis of clinical management of feline leishmaniosis caused by *Leishmania infantum*. Parasit Vectors. 2022;15:253. 10.1186/s13071-022-05369-6. PMC9272864 35818075

[24] Pennisi MG, Persichetti MF. Feline leishmaniosis: Is the cat a small dog? Vet Parasitol. 2018;251:131–7. 10.1016/j.vetpar.2018.01.012. PMC7130840 29426470

[25] Pennisi MG, Cardoso L, Baneth G, Bourdeau P, Koutinas A, Miró G, et al. LeishVet update and recommendations on feline leishmaniosis. Parasit Vectors. 2015;8:302. 10.1186/s13071-015-0909-z. PMC4462189 26041555

[26] Paltrinieri S, Gradoni L, Roura X, Zatelli A, Zini E. Laboratory tests for diagnosing and monitoring canine leishmaniasis. Vet Clin Pathol. 2016;45:552–78. 10.1111/vcp.12413. 27805725

[27] Pereira A, Mourão J, Cristóvão JM, Xufre Â, Maia C. Seroprevalence of *Leishmania* spp. infection in cats in Portugal. Microorganisms. 2026;14:668. 10.3390/microorganisms14030668. PMC13029042 41900427

[28] Villanueva-Saz S, Giner J, Marteles D, Verde M, Yzuel A, Riera C, et al. Leishmaniosis caused by *Leishmania infantum* in ferrets: Update review. Vet Anim Sci. 2021;15:100229. 10.1016/j.vas.2021.100229. PMC8739881 35028486

[29] Solano-Gallego L, Villanueva-Saz S, Carbonell M, Trotta M, Furlanello T, Natale A. Serological diagnosis of canine leishmaniosis: comparison of three commercial ELISA tests (Leiscan, ID Screen and *Leishmania* 96), a rapid test (Speed Leish K) and an in-house IFAT. Parasit Vectors. 2014;7:111. 10.1186/1756-3305-7-111. PMC3994334 24655335

[30] Iatta R, Carbonara M, Morea A, Trerotoli P, Benelli G, Nachum-Biala Y, et al. Assessment of the diagnostic performance of serological tests in areas where *Leishmania infantum* and *Leishmania tarentolae* occur in sympatry. Parasit Vectors. 2023;16:352. 10.1186/s13071-023-05981-0. PMC10561492 37807047

[31] Hermanson, 2013, Hermanson, G. T. Bioconjugate Techniques; Elsevier, 2013. 10.1016/B978-0-12-382239-0.00001-7.

[32] Foglia Manzillo V, Balestrino I, Oliva G, Brunetti R, Cavallo S, D’Ambrosio R, et al. High Seroprevalence of Feline Leishmaniosis (FeL) in Campania (Italy) Region: current epidemiological scenario. Pathogens. 2025;14:1194. 10.3390/pathogens14121194. PMC12735749 41471150

[33] Chatzis MK, Leontides L, Athanasiou LV, Papadopoulos E, Kasabalis D, Mylonakis M, et al. Evaluation of indirect immunofluorescence antibody test and enzyme-linked immunosorbent assay for the diagnosis of infection by *Leishmania infantum* in clinically normal and sick cats. Exp Parasitol. 2014;147:54–9. 10.1016/j.exppara.2014.10.004. PMC7094338 25307685

[34] Carbonara M, Iatta R, Miró G, Montoya A, Benelli G, Mendoza-Roldan JA, et al. Feline leishmaniosis in the Mediterranean basin: a multicenter study. Parasit Vectors. 2024;17:346. 10.1186/s13071-024-06419-x. PMC11331770 39160611

[35] Pennisi MG, Hartmann K, Lloret A, Addie D, Belák S, Boucraut-Baralon C, et al. Leishmaniosis in cats: ABCD guidelines on prevention and management. J Feline Med Surg. 2013;15:638–42. 10.1177/1098612X13489229. PMC11148954 23813831

[36] Rivas AK, Alcover MM, Martínez-Orellana P, Montserrat-Sangrà S, Nachum-Biala Y, Fisa R, et al. Serological and molecular survey of *Leishmania* infection in dogs from Venezuela. Vet Parasitol Reg Stud Reports. 2020;21:100420. 10.1016/j.vprsr.2020.100420. 32862890

[37] Giner J, González A, Lebrero ME, Quilez P, Guallar D, Gómez Á, et al. Changes in serological, inflammatory, and serum protein electrophoretic markers during ongoing meglumine antimoniate plus allopurinol therapy in canine leishmaniosis. Vet Res Commun. 2026;50:327. 10.1007/s11259-026-11275-4. PMC13179883 42142214

[38] Mahachi KG, Ozanne M, Bourdeau P, Sarquis J, Kontowicz E, Solano-Gallego L, et al. Comparison of ELISA and IFAT for *Leishmania infantum* by European and Middle Eastern diagnostic laboratories. Parasit Vectros. 2024;17:545. 10.1186/s13071-024-06631-9. PMC11684067 39734221

